# Integrated genomic, transcriptomic, and proteomic analyses prioritize cadmium-responsive class III peroxidases in *Salix variegata*

**DOI:** 10.3389/fpls.2026.1956650

**Published:** 2026-09-28

**Authors:** Renxiu Yao, Jian Li, Youwei Zuo, Nana Long, Hongping Deng

**Affiliations:** School of Life Sciences, Southwest University, Chongqing, China

**Keywords:** cadmium stress, class III peroxidase, DIA proteomics, gene family evolution, multi-omics integration, RNA sequencing, *Salix variegata*

## Abstract

Class III peroxidases (PRXs) link apoplastic redox regulation with cell-wall remodeling, yet their coordinated transcript- and protein-level responses to cadmium (Cd) remain poorly resolved in woody plants. We identified 80 high-confidence class III PRX genes in Salix variegata and characterized their genomic organization, evolutionary relationships, protein properties, and predicted regulatory features. Root RNA-seq and data-independent acquisition proteomic datasets from plants exposed to 0 or 100 μM CdCl_2_ for 10 d were then integrated to prioritize members supported across molecular layers. Segmental and dispersed duplication were the predominant expansion modes, and most SvaPRX proteins showed sequence features compatible with extracellular localization. RNA-seq identified 42 upregulated and two downregulated SvaPRX genes. The DIA dataset comprised 22,883 peptides and 6,068 proteins, with 4,849 proteins quantified in all six samples; biological replicates showed high within-treatment correlations and separation of CK and Cd100 in principal component analysis. Forty SvaPRX proteins were quantified, including 11 upregulated and six downregulated proteins. Cross-layer integration reduced the broad transcript-responsive set to seven concordantly upregulated candidates: SvPRX12, SvPRX23, SvPRX41, SvPRX47, SvPRX61, SvPRX62, and SvPRX66. Targeted qRT-PCR provided additional directional support for Cd-induced expression of SvPRX61 and SvPRX62, with response directions consistent across RNA-seq, proteomic, and qRT-PCR datasets. These results establish a reproducible genome-to-proteome framework for prioritizing Cd-responsive class III peroxidases and identify focused candidates for subsequent biochemical and genetic investigation in woody plants.

## 1 Introduction

Cadmium (Cd) is a non-essential and highly toxic metal that can be readily absorbed by plant roots and subsequently transported to aerial tissues. Excess Cd inhibits root development, disrupts mineral-nutrient homeostasis, impairs photosynthesis and primary metabolism, and ultimately restricts plant growth and biomass production (Haider et al., 2021; Huybrechts et al., 2019). Although Cd is not directly redox-active, its accumulation disturbs cellular redox balance and promotes the excessive production of reactive oxygen species (ROS), leading to lipid peroxidation, protein oxidation, nucleic-acid damage, and membrane dysfunction (Huybrechts et al., 2019; Cuypers et al., 2010). Plants therefore rely on coordinated responses involving metal uptake and transport, chelation and sequestration, antioxidant regulation, and maintenance of cellular structural integrity to alleviate Cd toxicity (Haider et al., 2021; Huybrechts et al., 2019; Cuypers et al., 2010).

The plant cell wall represents both the first cellular barrier encountered by external Cd and an important compartment for metal immobilization. Functional groups in pectin, hemicellulose, lignin, and cell-wall-associated proteins can bind metal ions and reduce their entry into the symplast (Krzesłowska, 2011; Parrotta et al., 2015). Cd exposure can also alter cell-wall polysaccharide composition, lignification, cross-linking, and apoplastic redox conditions (Krzesłowska, 2011; Parrotta et al., 2015; Loix et al., 2017). These processes are closely connected with ROS metabolism because hydrogen peroxide is required for the oxidative polymerization of lignin and the cross-linking of cell-wall components. Conversely, excessive ROS accumulation can damage cellular membranes and disturb normal cell-wall development. Coordination between apoplastic ROS regulation and cell-wall remodeling may therefore represent an important component of plant adaptation to Cd stress (Parrotta et al., 2015; Loix et al., 2017).

Class III peroxidases (PRXs; EC 1.11.1.7) are plant-specific, heme-containing enzymes that are generally secreted into the apoplast or associated with the cell wall (Shigeto and Tsutsumi, 2016; Cosio and Dunand, 2009; Francoz et al., 2015). They use hydrogen peroxide to oxidize a broad range of phenolic and non-phenolic substrates and participate in lignin and suberin polymerization, cell-wall cross-linking, auxin metabolism, cell elongation, root development, senescence, and responses to environmental stresses (Shigeto and Tsutsumi, 2016; Cosio and Dunand, 2009; Francoz et al., 2015). Depending on the catalytic cycle, substrate availability, and physiological context, class III PRXs may either consume hydrogen peroxide or contribute to the generation of reactive oxygen intermediates (Shigeto and Tsutsumi, 2016; Cosio and Dunand, 2009). Functional studies of individual PRXs have further demonstrated their diverse roles in ROS distribution, cell-wall integrity, root-hair development, and stress-associated lignification (Raggi et al., 2015; Marzol et al., 2022; Pacheco et al., 2022; Kidwai et al., 2019). This catalytic and functional versatility makes class III PRXs plausible molecular links between oxidative regulation and cell-wall-associated responses during metal stress.

Class III PRXs are usually encoded by large multigene families. Although their proteins share a conserved catalytic framework, individual members may differ considerably in gene structure, protein length, signal peptides, subcellular localization, promoter composition, expression pattern, and substrate preference (Tognolli et al., 2002; Welinder et al., 2002; Wang et al., 2015). Whole-genome analyses in *Arabidopsis thaliana* and maize have shown that duplication and subsequent sequence or regulatory divergence contributed to PRX-family expansion and functional diversification (Tognolli et al., 2002; Welinder et al., 2002; Wang et al., 2015). However, stress-responsive changes in transcript abundance are not necessarily accompanied by corresponding changes in protein abundance. Post-transcriptional regulation, translation efficiency, protein turnover, and secretion can all contribute to differences between transcriptomic and proteomic responses. Integrating RNA-seq and proteomic evidence can therefore refine the identification of stress-responsive candidates and reduce reliance on transcript-level responses alone (Zhu et al., 2022).

Willows (*Salix* spp.) provide a useful woody system for investigating heavy-metal responses because their rapid growth, extensive root systems, high biomass production, ease of vegetative propagation, and ability to coppice can be combined with appreciable metal uptake in several species and genotypes. In phytomanagement applications, high biomass production is particularly important because total metal removal depends on both tissue metal concentration and harvestable biomass. Field and controlled-environment studies have demonstrated that selected willow genotypes can maintain substantial biomass production on metal-contaminated substrates and accumulate or translocate Cd, although these capacities vary markedly among genotypes (Van Slycken et al., 2013; Song et al., 2023). The fast growth of willows also requires sustained root development, cell-wall expansion, lignification, and redox regulation—processes in which class III PRXs participate. Characterizing Cd-responsive PRXs may therefore help clarify how cell-wall and apoplastic redox processes are coordinated with growth-related functions in a fast-growing woody plant, without implying that individual PRXs have already been demonstrated to confer Cd tolerance or phytoremediation capacity.

*Salix variegata* is a native riparian shrub of the Three Gorges Reservoir region with a well-developed root system and the capacity to grow on barren gravel riverbanks and tolerate inundation and water-flow disturbance; it has consequently been used in regional riparian shelter vegetation (Ayi et al., 2016). Previous hydroponic studies have further shown that *S. variegata* takes up Cd and exhibits Cd-dependent changes in root activity and mineral-element homeostasis (Liu et al., 2016), while other studies have implicated antioxidant regulation, secondary metabolism, and cell-wall-associated processes in its Cd response. Together with the availability of a chromosome-scale genome (Zhang et al., 2025), these ecological, physiological, and genomic attributes make *S. variegata* a relevant non-model woody species for family-wide and cross-omics analysis of Cd-responsive class III PRXs.

We hypothesized that a subset of the S. variegata class III PRX family would show coordinated transcriptomic and proteomic responses to Cd stress and could therefore be prioritized more robustly than by transcript data alone. We first identified and characterized the family at genomic, evolutionary, structural, protein, and promoter levels. Root RNA-seq and DIA proteomic datasets obtained under CK and Cd100 conditions were then integrated to distinguish concordant, discordant, and single-layer responses. Seven genes with significant and directionally concordant increases at both molecular levels were retained as Tier 1 candidates, and SvPRX61 and SvPRX62 were subjected to a targeted qRT-PCR assessment. The study was designed to provide an experimentally anchored, family-specific prioritization framework rather than to assign physiological functions to individual PRX genes.

## 2 Materials and methods

### 2.1 Plant materials and cadmium treatment

Uniform two-month-old *Salix variegata* Franch. plants were obtained from a germplasm resource area along the Jialing River in Beibei District, Chongqing, China (106.42° E, 29.85° N). The plants were cultivated in 0.7-L plastic containers containing half-strength Hoagland nutrient solution, with three plants per container. The plants were maintained under controlled-environment conditions of 25 °C during the light period, 20 °C during the dark period, a 16-h light/8-h dark photoperiod, and an approximate illuminance of 6000 lx. This moderate artificial-light regime was selected to maintain uniform growth of the hydroponically cultured plants while minimizing additional high-light stress during Cd exposure. Comparable controlled conditions, including a 16-h photoperiod and an irradiance of 95 μmol m^-^² s^-^¹, have previously been used for hydroponically cultured *S. variegata* (Zhang et al., 2020). The imposed light condition was intended to provide a reproducible controlled environment rather than to simulate full natural sunlight.

Plants were exposed to nutrient solution containing either 0 μM CdCl_2_ (CK) or 100 μM CdCl_2_ (Cd100) for 10 d. The Cd100 treatment corresponds to approximately 11.24 mg Cd L^-^¹. This concentration was used as a defined stress-inducing treatment to elicit a measurable root molecular response for transcriptome–proteome integration rather than to reproduce typical dissolved Cd concentrations in natural environments. The selected concentration lies within the range previously examined in hydroponically cultured S. variegata: Liu et al. (2016) tested 2–50 mg Cd L^-^¹ and detected a significant reduction in root activity at concentrations of 10 mg L^-^¹ and above, while Zhang et al. (2020) used 50 μM Cd to investigate physiological, antioxidant, and gene-expression responses in this species. The nutrient solution was renewed every 3 d. At the end of the treatment, roots were harvested, immediately frozen in liquid nitrogen, and stored at −80 °C. For each RNA-seq and proteomic biological replicate, roots from the three plants within one container were pooled. Three independent containers were analyzed per treatment. Three independent root biological replicates were used for the targeted qRT-PCR analysis.

### 2.2 Identification and protein characterization of the SvaPRX family

The chromosome-scale genome assembly, predicted protein sequences, and genome annotation of *S. variegata* were obtained from assembly GWHEUCC00000000.1 under BioProject PRJCA026796 (Zhang et al., 2025). Seventy-three curated class III peroxidase proteins from *Arabidopsis thaliana* were used as reference sequences.

The reference proteins were searched against the predicted *S. variegata* proteome using BLASTP in NCBI BLAST+ v2.17.0 (Camacho et al., 2009). Searches were performed using an E-value threshold of 1 × 10^-5^, a maximum of 100 target sequences per query, and one high-scoring segment pair per query-target combination. Preliminary candidates were retained when they showed an E-value ≤ 1 × 10^-5^, amino-acid identity ≥ 30%, alignment length ≥ 100 aa, and query coverage ≥ 50%.

Candidate proteins were further examined using the NCBI Conserved Domain Database, Pfam, and InterPro (Lu et al., 2020; Mistry et al., 2021; Paysan-Lafosse et al., 2023). Candidates showing coherent class III or secretory peroxidase support from CDD cd00693, Pfam PF00141, and/or InterPro entries IPR000823 and IPR033905 were retained after manual integration of the annotation results. Proteins annotated as class I or ascorbate-peroxidase-like proteins were excluded. One additional candidate lacking sufficient Sec/SPI and secretory-peroxidase support was removed during manual curation. The final family comprised 80 high-confidence class III peroxidase genes, designated *SvPRX01*–*SvPRX80* according to their genomic positions.

Protein length was calculated directly from the amino-acid sequences. Molecular weight and theoretical isoelectric point were calculated using the Peptides package in R (Osorio et al., 2015). N-terminal signal peptides were predicted using SignalP 6.0 with the Eukarya model (Teufel et al., 2022). Subcellular localization and transmembrane topology were predicted using DeepLoc 2.1 and DeepTMHMM, respectively (Thumuluri et al., 2022; Hallgren et al., 2022). Default settings were used unless otherwise stated. These results were treated as sequence-based predictions rather than experimental localization evidence.

### 2.3 Phylogenetic, chromosomal, duplication, gene-structure, and motif analyses

Full-length amino-acid sequences of the 80 SvaPRX proteins and 73 *A. thaliana* class III peroxidases were aligned using MAFFT v7 (Katoh and Standley, 2013). A maximum-likelihood phylogenetic tree was constructed using IQ-TREE v3.1.3 with the options -m MFP -alrt 1000 -B 1000 -nt AUTO. The optimal amino-acid substitution model was selected automatically using ModelFinder, and branch support was evaluated using 1000 SH-like approximate likelihood-ratio test replicates and 1000 ultrafast bootstrap replicates (Wong et al., 2026; Kalyaanamoorthy et al., 2017; Hoang et al., 2018). The consensus tree was visualized and annotated using iTOL (Letunic and Bork, 2021). Based on the topology of the combined tree, SvaPRX members were assigned to eight study-defined phylogenetic groups, C1–C8, or to the heterogeneous Other category.

Chromosomal positions, gene coordinates, and strand information were extracted from the *S. variegata* GFF3 annotation. Whole-proteome self-alignment was performed using DIAMOND in sensitive mode with an E-value threshold of 1 × 10^-^¹^0^ and a maximum of five target sequences per query (Buchfink et al., 2021). Within-genome collinearity and duplication patterns were analyzed using MCScanX with a minimum collinear-block size of five genes and a maximum of 20 gaps (Wang et al., 2012). The duplicate_gene_classifier program was used to classify genes as segmental, dispersed, tandem, proximal, or singleton duplicates. Physical proximity alone was not considered evidence of tandem duplication unless supported by the MCScanX classification.

Exon, coding-sequence, and untranslated-region coordinates were extracted from the genome annotation and normalized to the 5′–3′ orientation. Conserved protein motifs were identified using MEME Suite v5.5.9 in protein mode with the zero-or-one-occurrence-per-sequence model, a maximum of 10 motifs, and motif widths ranging from 6 to 50 amino acids (Bailey et al., 2015). All 80 SvaPRX proteins were included in the complete analysis, whereas the main structural figure displayed members assigned to the resolved C1–C8 groups.

### 2.4 Promoter extraction and predicted regulatory-motif analysis

For each SvPRX gene, up to 2000 bp upstream of the annotated 5′ gene boundary was extracted in a strand-aware manner. When the complete 2-kb upstream interval was unavailable because of a reference-sequence boundary, the available sequence was retained and recorded as boundary-truncated.

Potential transcription-factor-binding motifs were identified using FIMO in MEME Suite v5.5.9 and the JASPAR 2026 CORE plants non-redundant motif collection (Grant et al., 2011; Ovek Baydar et al., 2026). Predicted motif hits with a Benjamini–Hochberg-adjusted q value ≤ 0.05 were retained. Predicted motif-hit density was expressed as hits per kilobase of the actual promoter sequence:

The number of unique predicted motifs was also calculated for each promoter. These analyses were used to describe promoter-sequence characteristics and were not interpreted as evidence of transcription-factor binding, motif enrichment, or direct transcriptional regulation. The final dataset contained 79 complete 2-kb promoters and one boundary-truncated promoter.

### 2.5 RNA sequencing and expression analysis

Total RNA was extracted from three CK and three Cd100 root biological samples using TRIzol Reagent (Invitrogen, Carlsbad, CA, USA). Residual genomic DNA was removed using DNase I (TaKaRa, Dalian, China). RNA integrity and purity were assessed using an Agilent 2100 Bioanalyzer and a NanoDrop ND-2000 spectrophotometer. Strand-specific mRNA libraries were prepared using the TruSeq RNA Sample Preparation Kit and sequenced on an Illumina NovaSeq 6000 platform to generate 150-bp paired-end reads.

Raw reads were filtered using Trimmomatic (Bolger et al., 2014) and aligned to the *S. variegata* reference genome using HISAT2 (Kim et al., 2019). Gene-level read assignment and alignment-quality assessment were performed using HTSeq and Qualimap, respectively (Anders et al., 2015; Okonechnikov et al., 2016). Differential expression between Cd100 and CK was analyzed using edgeR (Robinson et al., 2010). Genes with FDR < 0.05 and |log_2_ fold change| ≥ 1 were considered differentially expressed.

Gene-level TPM values were used to evaluate transcript abundance, calculate sample correlations, and generate the full-family expression heatmap. For heatmap visualization, log_2_(TPM + 1) values were standardized to row Z-scores. Differential-expression status was obtained from the formal CK-versus-Cd100 DEG dataset and was not recalculated from TPM values. Genes not identified as DEGs under the applied criteria were retained in the family-wide TPM and cross-layer analyses but were not assigned a significant RNA-seq response.

### 2.6 DIA quantitative proteomic analysis and quality assessment

Three independent CK and three independent Cd100 root samples were subjected to DIA quantitative proteomic analysis. Frozen roots were ground under liquid nitrogen and extracted using SDT buffer containing 100 mM NaCl and dithiothreitol. Samples were sonicated in an ice-water bath for 5 min, heated at 95 °C for 8–15 min, and centrifuged at 12,000 × g for 15 min at 4 °C. Proteins in the supernatant were alkylated with iodoacetamide for 1 h in darkness and precipitated using four volumes of precooled acetone at −20 °C for at least 2 h. The protein pellets were washed with precooled acetone and dissolved in buffer containing 8 M urea and 100 mM triethylammonium bicarbonate at pH 8.5.

Protein concentrations were determined using the Bradford assay, and protein quality was evaluated by SDS-PAGE. Proteins were initially digested with trypsin at 37 °C for 4 h, followed by an additional overnight digestion. The resulting peptides were acidified with formic acid, desalted using C18 cartridges, and lyophilized.

Approximately 200 ng of peptides per sample was separated using a Vanquish Neo nano-UHPLC system equipped with a C18 trap column and a PepMap Neo C18 analytical column. Peptides were analyzed using an Orbitrap Astral mass spectrometer with an electrospray voltage of 1.9 kV and an ion-transfer-tube temperature of 290 °C. Data were acquired in DIA mode. The MS1 scan range was m/z 380–980 at a resolution of 240,000. A total of 300 consecutive 2-Th isolation windows were used, with a normalized collision energy of 25%, a fragment-ion range of m/z 150–2000, and a maximum injection time of 3 ms.

Raw files were processed using DIA-NN against the project protein database (Demichev et al., 2020). Precursor-level and protein-group identifications were retained at global q values below 0.01. Differentially abundant proteins between Cd100 and CK were identified in the supplied project-level differential-abundance table using FDR ≤ 0.05 together with a ≥1.5-fold increase or ≤0.667-fold decrease. For global proteomic quality assessment, positive protein-abundance values were log_2_-transformed. The primary principal component analysis used the 4,849 proteins quantified in all six samples. To assess whether complete-case filtering materially affected treatment separation, a sensitivity PCA was also performed using 5,921 proteins quantified in at least three of the six samples; missing log_2_ abundances were replaced by the median of the observed log_2_ abundances for the corresponding protein before centering, and no variance scaling was applied. Pairwise Pearson correlations were calculated from log_2_-transformed abundances using proteins quantified in both samples. Within-treatment coefficients of variation were calculated separately for CK and Cd100 using proteins quantified in all three biological replicates of the corresponding treatment.

### 2.7 Integration of RNA-seq and proteomic evidence

RNA-seq and proteomic records were matched to the SvaPRX family using gene and protein identifiers. Formal DEG and DEP results were used to distinguish directionally concordant RNA–protein responses, discordant responses, RNA-seq-only responses, proteome-only responses, and genes lacking joint formal differential evidence.

When multiple significant protein records mapped to the same SvPRX gene, a gene-level protein direction was assigned only when the retained records showed a consistent direction of change. The median record-level protein log_2_ fold change was used for descriptive visualization, and no new gene-level proteomic FDR was calculated.

The seven genes showing significant and directionally concordant increases at both molecular levels—SvPRX12, SvPRX23, SvPRX41, SvPRX47, SvPRX61, SvPRX62, and SvPRX66—were retained as Tier 1 candidates. Tier membership was determined by the cross-layer significance and direction criteria, not by a weighted functional score. For reproducible display only, candidates were ordered using RNA-seq FDR, proteomic FDR, absolute RNA-seq and protein effect sizes, Cd100 transcript abundance, and gene name as sequential tie-breakers. Protein-level responses, promoter characteristics, phylogenetic placement, and duplication origin were retained as contextual evidence and were not used to infer function.

Only SvPRX61 and SvPRX62 were subsequently examined by qRT-PCR. They were selected as representative high-priority candidates because SvPRX62 and SvPRX61 ranked first and third, respectively, in the reproducible Tier 1 display order, showed concordant induction at the transcript and protein levels, represented different phylogenetic contexts (C7 and Other), and yielded gene-specific, reproducible qRT-PCR assays. The remaining five genes were retained as Tier 1 cross-layer candidates but were not examined by qRT-PCR; the targeted two-gene assay was therefore interpreted as additional directional support for these two genes rather than validation of the Tier 1 set as a whole.

### 2.8 qRT-PCR analysis and statistical assessment

Total RNA was extracted from three independent CK and three independent Cd100 root biological replicates. First-strand cDNA was synthesized from 1 μg of total RNA using SynScript III One-Tube RT SuperMix with gDNA Remover (Tsingke Biotechnology, Beijing, China; DLR102). Each 20-μL reverse-transcription reaction contained 5 μL of 4× One-Tube RT SuperMix, RNA template, and RNase-free water. Reverse transcription was performed at 37 °C for 2 min, 55 °C for 10 min, and 92 °C for 5 s.

qRT-PCR was performed using ArtiCan SYBR qPCR Mix (Tsingke Biotechnology; DLQ102) without ROX reference dye. Each 10-μL reaction contained 5.0 μL of 2× SYBR qPCR mix, 0.4 μL of each 10 μM primer, 2.0 μL of cDNA, and 2.2 μL of RNase-free water. Amplification was performed at 95 °C for 1 min, followed by 40 cycles of 95 °C for 10 s and 60 °C for 20 s. Melting-curve analysis was performed after amplification. Each biological sample was analyzed using three technical replicates.

TIP41 (Sva09G007630) was used as the working reference gene. Its selection was supported by a systematic evaluation of 14 candidate reference genes in the related willow Salix viminalis, in which TIP41 and CDC2 were the most stable genes in roots across multiple abiotic-stress conditions, including a poly-metal treatment (Ambroise et al., 2019). As an orthogonal check in the present dataset, Sva09G007630 showed mean TPM values of 28.38 in CK and 33.49 in Cd100 roots, corresponding to a modest descriptive log_2_ ratio of 0.239, and was not present in the supplied significant-DEG list. This RNA-seq observation was treated as supporting information rather than a formal multi-reference-gene qPCR stability assessment. Relative expression was calculated using the 2^-^ΔΔCq method, with CK used as the calibrator (Livak and Schmittgen, 2001). Primer sequences and amplicon lengths for SvPRX61, SvPRX62, and TIP41 are provided in **Supplementary Table S11**. Technical replicates were first examined for amplification quality and averaged within each biological replicate. Biological replicates were treated as independent experimental units. Statistical comparisons between CK and Cd100 were performed separately for each target gene using biological-replicate ΔCq values and Welch’s two-sample t-test, rather than the transformed relative-expression values. Data are presented as the mean ± standard deviation, and P < 0.05 was considered statistically significant. Data integration, statistical analysis, and figure preparation were performed using R v4.5.1.

## 3 Results

### 3.1 Identification, phylogenetic classification, and protein characteristics

A total of 73 class III peroxidase proteins from *Arabidopsis thaliana* were used to search the predicted proteome of *Salix variegata*. The BLASTP search generated 6376 hits corresponding to 91 non-redundant *S. variegata* proteins. Domain-based screening retained 89 proteins with peroxidase-domain support. Following integrated examination of conserved-domain annotations, signal-peptide predictions, and manual-curation records, eight class I/ascorbate-peroxidase-like proteins and one candidate lacking sufficient secretory-peroxidase support were excluded. The final family therefore comprised 80 high-confidence class III peroxidases, designated SvaPRX01–SvaPRX80 according to their genomic positions (**Supplementary Figure S1**; **Supplementary Tables S1**, **S2**).

Phylogenetic relationships were examined using the 80 SvaPRX proteins together with the 73 *A. thaliana* class III peroxidases. Eight non-overlapping clades were selected from the topology of the combined maximum-likelihood tree and designated C1–C8. These labels were defined in the present study as phylogenetic units for comparative analysis and were not intended to correspond directly to previously proposed functional subclasses of plant class III peroxidases. A total of 46 SvaPRX proteins were assigned to these groups, including 11 members in C1, nine in C2, eight in C3, five in C4, four in C5, and three members each in C6, C7, and C8 (**Figure 1**).

**Figure 1:**
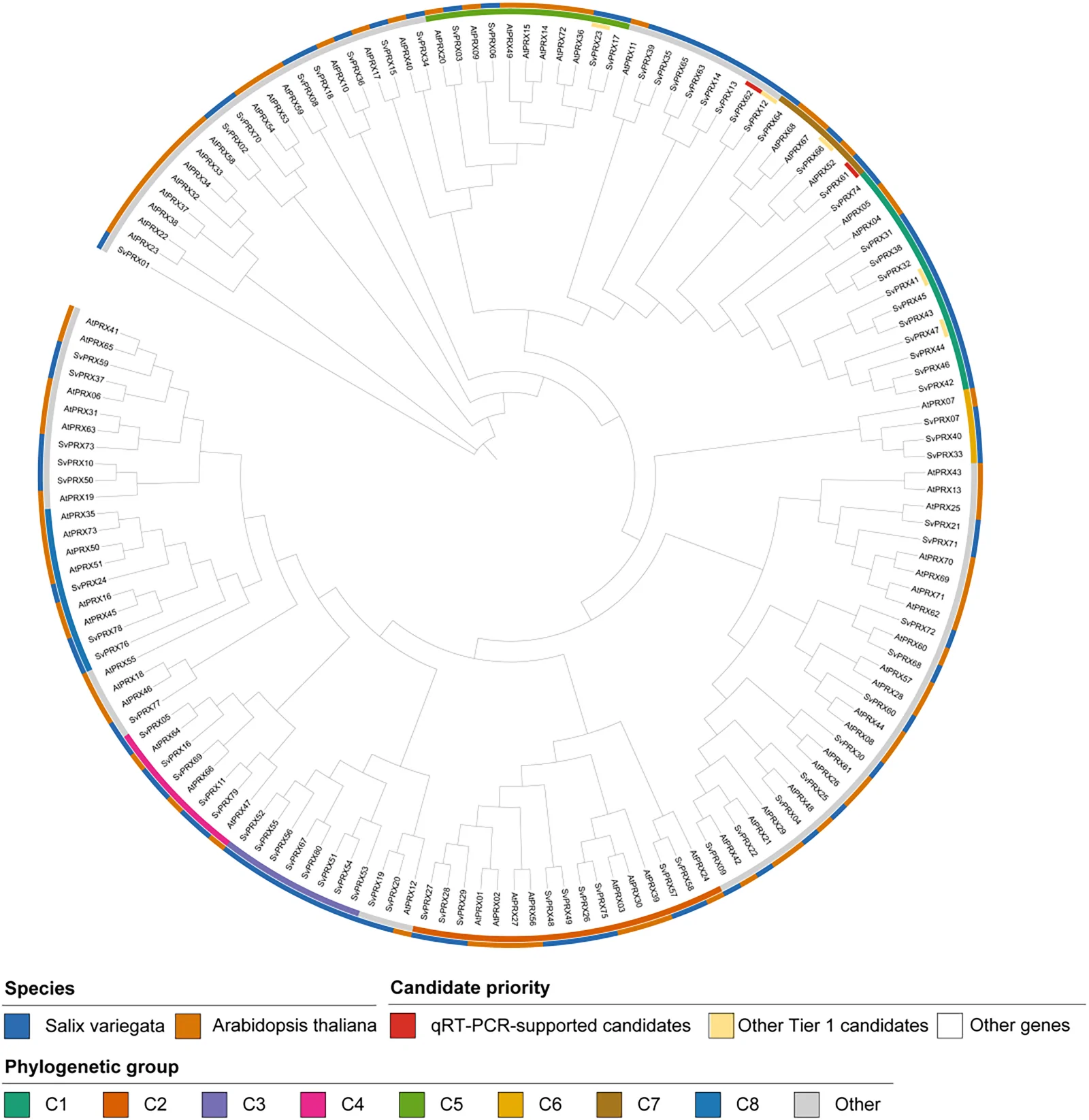
Phylogenetic relationships of 80 SvaPRX proteins and 73 Arabidopsis thaliana class III peroxidases. The circular maximum-likelihood tree was constructed from full-length protein sequences. Outer annotation rings indicate species, phylogenetic group (C1–C8 and Other), and candidate priority. SvPRX61 and SvPRX62 are designated as qRT-PCR-supported candidates, whereas SvPRX12, SvPRX23, SvPRX41, SvPRX47, and SvPRX66 are designated as other Tier 1 candidates.

The remaining 34 proteins did not fall within the eight selected clades and were retained as a heterogeneous Other category. This designation did not indicate low sequence confidence or reduced biological importance, and all Other members were included in the subsequent structural, transcriptomic, proteomic, and candidate-prioritization analyses. Notably, *SvPRX62*, one of the two genes subsequently examined by qRT-PCR, belonged to this category, whereas *SvPRX61* was assigned to C7. The other five Tier 1 candidates were distributed among C1, C5, C7, and Other. The tree-tip order, species information, phylogenetic assignments, and final candidate categories used to annotate **Figure 1** are provided in **Supplementary Table S4**.

The predicted SvaPRX proteins ranged from 272 to 741 amino acids in length, with a median length of 328 amino acids. DeepLoc predicted 72 proteins to be extracellular, whereas five were assigned to the lysosome/vacuole, two to plastids, and one to the peroxisome. Integration of SignalP, DeepLoc, and DeepTMHMM predictions provided consistent secretory–extracellular support for 65 proteins. Seven additional proteins were predicted to be extracellular despite lacking a SignalP-supported signal peptide, and one protein was flagged for potential transmembrane localization (**Supplementary Figure S2**). These results indicate that most members possess sequence features compatible with secretion or extracellular localization, although these predictions require experimental confirmation. Detailed protein properties and localization predictions for all 80 members are provided in **Supplementary Table S3**.

### 3.2 Chromosomal distribution and duplication patterns

The 80 SvPRX genes were unevenly distributed throughout the *S. variegata* genome. Seventy-nine genes were located on the 19 chromosomes, whereas one gene was located on an unplaced genomic sequence. Chromosome 10 contained the largest number of family members, with 11 genes, followed by chromosome 12 with nine genes and chromosome 7 with eight genes. In contrast, chromosomes 9, 14, and 19 each contained only one SvPRX gene (**Figure 2A**). Chromosomal positions and duplication classifications for individual SvaPRX genes are provided in **Supplementary Table S5**.

**Figure 2:**
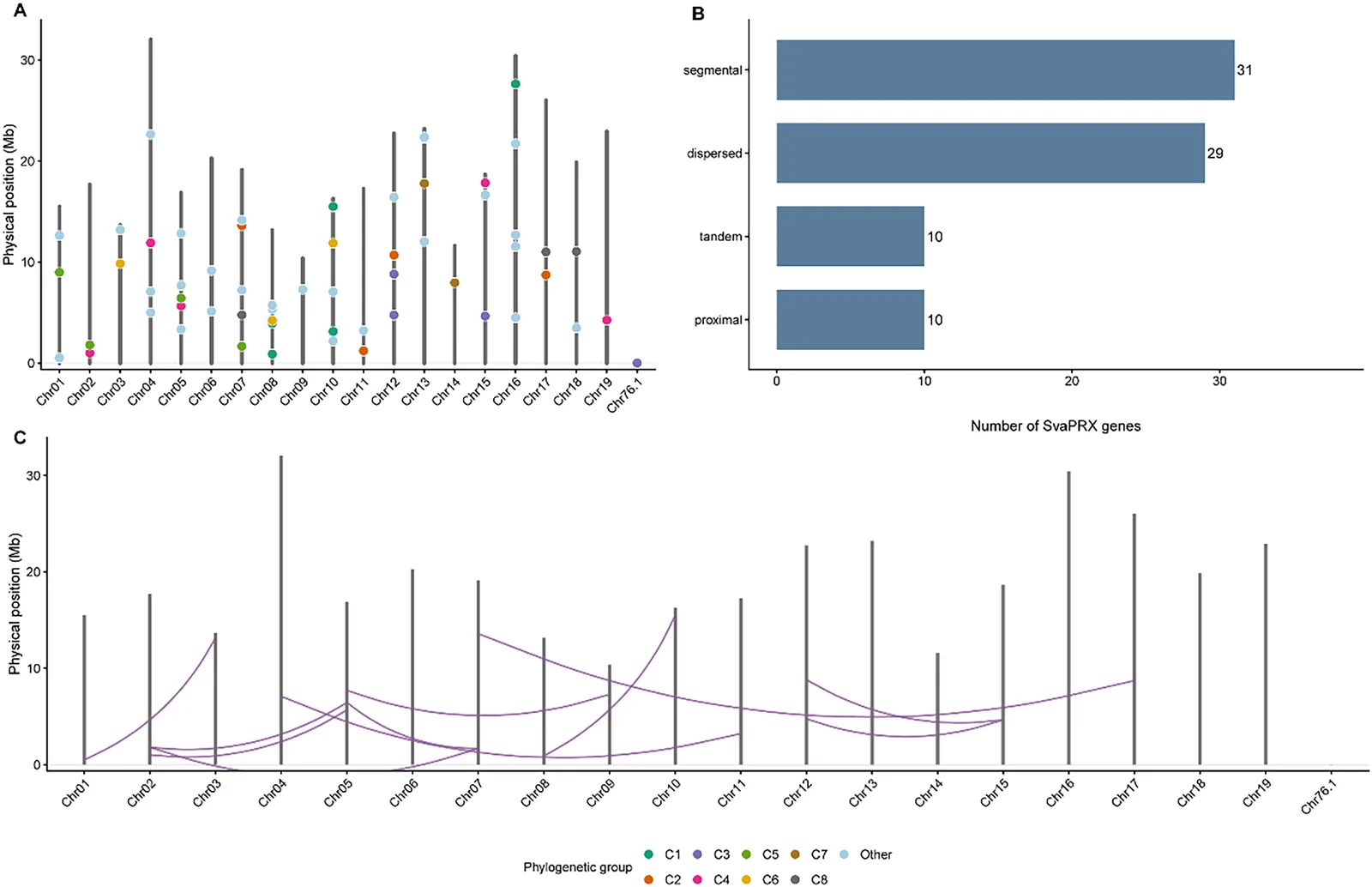
Chromosomal distribution and duplication patterns of the SvaPRX family. **(A)** Physical positions of SvaPRX genes on the *S. variegata* chromosomes and the unplaced genomic sequence. Point colors indicate phylogenetic groups. **(B)** Numbers of SvaPRX genes assigned to different duplication classes. **(C)** Direct segmental-collinearity relationships among duplicated SvaPRX loci.

Duplication-origin analysis assigned 31 genes to the segmental category, 29 to the dispersed category, and ten genes each to the tandem and proximal categories. No member was classified as a singleton (**Figure 2B**). Eleven direct segmental-collinearity relationships involving 18 SvPRX genes were identified across different chromosomes (**Figure 2C**). Several genes participated in more than one relationship; for example, *SvPRX06* was linked to *SvPRX17* and *SvPRX23*, whereas *SvPRX17* was also linked to *SvPRX23*. These results indicate that both large-scale duplication and local duplication contributed to the expansion of the SvaPRX family. The complete list of MCScanX-supported collinear SvaPRX gene pairs is provided in **Supplementary Table S6**.

### 3.3 Gene structures, conserved motifs, and promoter characteristics

Gene structures and conserved protein motifs were examined to evaluate structural conservation and diversification within the family. The number of exons ranged from one to 15. Four-exon structures were the most frequent and occurred in 44 genes, whereas 17 genes contained three exons. Thus, 61 of the 80 family members contained either three or four exons. Closely related members generally showed similar exon–intron arrangements, although several genes encoding unusually long proteins displayed more complex structures (**Figure 3**; **Supplementary Figure S3**).

**Figure 3:**
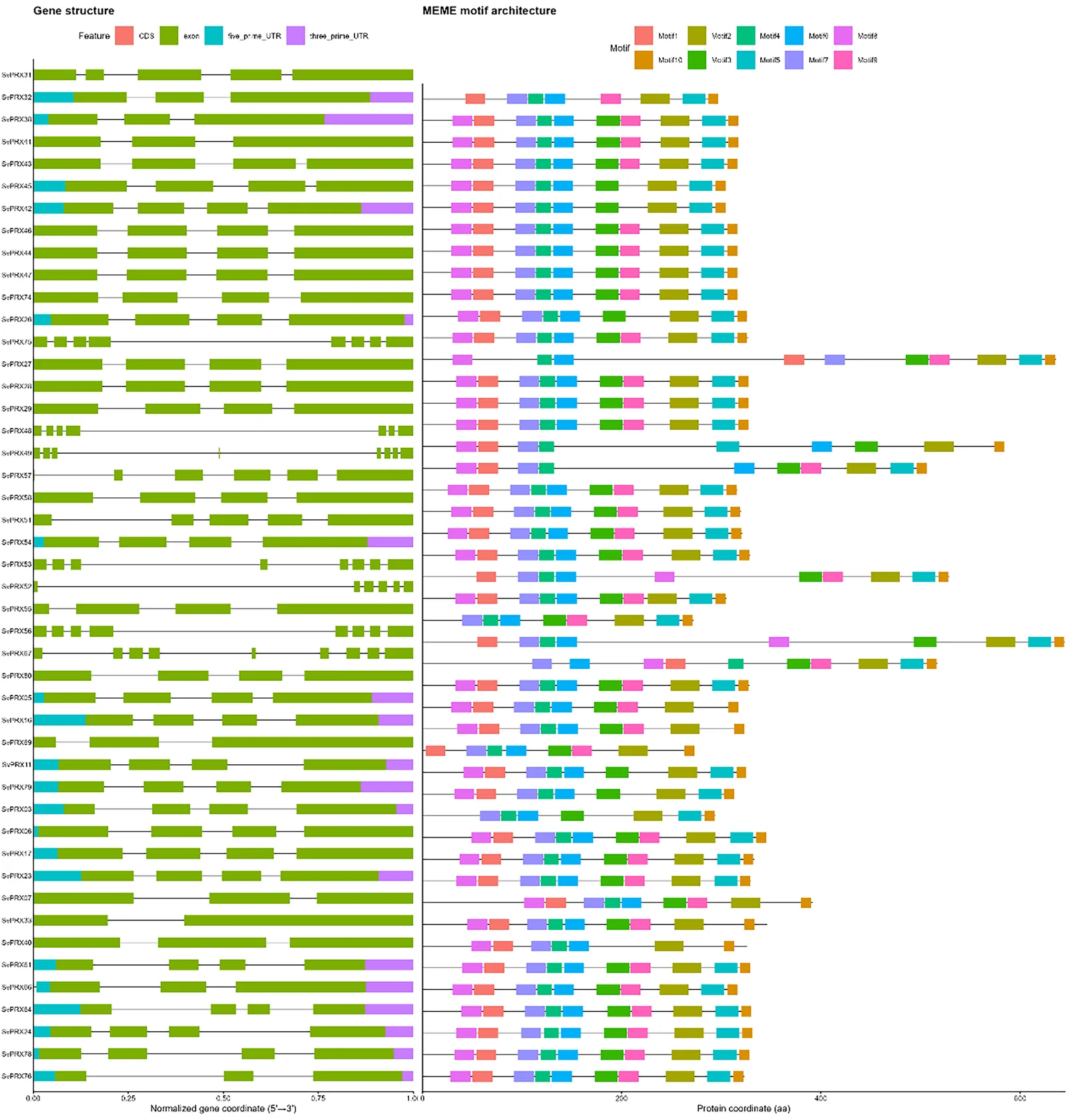
Gene structure and conserved motif organization of SvaPRX members from the selected C1-C8 phylogenetic groups. Exon-intron structures are shown on the left, and the positions of conserved MEME motifs in the encoded proteins are shown on the right. The complete analysis of all 80 SvaPRX members is provided in **Supplementary Figure S3**.

Ten conserved protein motifs were identified, comprising 759 motif sites across the 80 SvaPRX proteins. Motifs 2, 4, 6, and 7 were present in all 80 members. Motifs 10 and 1 occurred in 79 and 78 proteins, respectively, whereas Motifs 3, 5, 8, and 9 were detected in 75, 73, 74, and 60 proteins, respectively. The widespread occurrence of most motifs supports the overall conservation of the family, whereas the absence or altered arrangement of several less widely distributed motifs indicates sequence diversification among individual members. Motif positions for all 80 proteins are shown in **Supplementary Figure S3**, and detailed exon–intron, coding-sequence, UTR, and conserved-motif information is provided in **Supplementary Table S7**.

Complete 2-kb upstream sequences were obtained for 79 SvPRX genes, whereas the available upstream region of *SvPRX80* was truncated at a genomic-sequence boundary. FIMO analysis against the JASPAR plant collection identified 13,522 predicted motif sites representing 57 unique motifs. The number of predicted sites ranged from 11 to 544 per promoter, with a median of 159. DOF3.6 and AHL13 were the most frequently detected motifs and occurred in all 80 promoter sequences.

Substantial variation was observed among individual genes and phylogenetic groups in both predicted motif density and motif diversity (**Supplementary Figure S4**). Because the analysis was based on motif scanning without a formal enrichment comparison against a background promoter set, these results were interpreted as descriptive regulatory-sequence characteristics rather than evidence of Cd-responsive transcription-factor binding. The promoter features were subsequently retained as supporting contextual information during candidate comparison. Promoter-sequence metadata, retained FIMO hits, predicted motif-hit densities, and motif-diversity statistics are provided in **Supplementary Table S8**.

### 3.4 Cd-responsive transcriptional patterns

Expression patterns of the SvaPRX family were examined using root RNA-seq data from three CK and three Cd100 biological samples. Pearson correlation coefficients among the six libraries ranged from 0.887 to 0.985, indicating a generally consistent expression structure among the biological replicates (**Supplementary Figure S5A**). The full-family heatmap showed clear differences between CK and Cd100 samples for multiple SvaPRX genes (**Supplementary Figure S5B**). Per-sample TPM values, mean transcript abundances, RNA-seq log_2_ fold changes, FDR values, and Cd-responsive classifications for all SvaPRX genes are provided in **Supplementary Table S9**.

Among the 80 genes, 42 were significantly upregulated and two were significantly downregulated under Cd100 treatment, whereas 36 were not identified as differentially expressed under the applied criteria (**Supplementary Figure S5C**). The proportions of responsive genes differed among the phylogenetic groups. All members of C7 and C8 were upregulated, and high proportions of upregulated genes were also observed in C1, C3, and C5. The two downregulated genes were assigned to C1 and C4 (**Figure 4A**).

**Figure 4:**
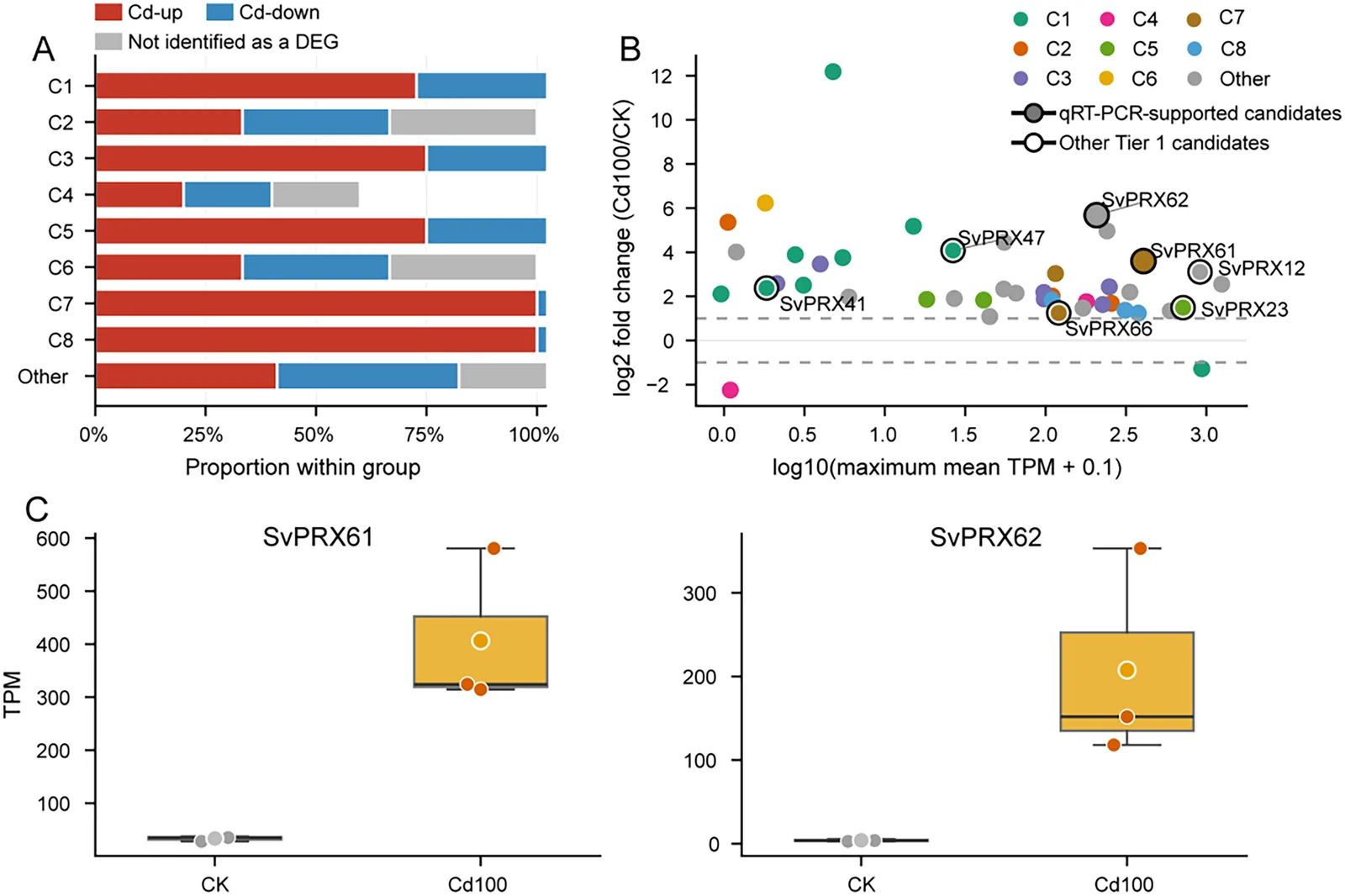
Cadmium-responsive transcriptional patterns of the SvaPRX family. **(A)** Proportions of Cd-upregulated DEGs, Cd-downregulated DEGs, and genes not identified as DEGs within each phylogenetic group based on the CK-versus-Cd100 differential-expression analysis. **(B)** Gene-level RNA-seq responses plotted as log_2_ fold change against log_10_(maximum mean TPM + 0.1). Point colors indicate phylogenetic groups. Filled points with black outlines indicate the qRT-PCR-supported candidates SvPRX61 and SvPRX62, whereas open points with black outlines indicate the other five Tier 1 candidates. Dashed lines indicate |log_2_ fold change| = 1. **(C)** Root RNA-seq TPM distributions of SvPRX61 and SvPRX62 in CK and Cd100 samples across three biological replicates.

Gene-level comparison of transcript abundance and effect size revealed substantial variation among the Cd-responsive members (**Figure 4B**). All seven Tier 1 candidates were transcriptionally upregulated. *SvPRX62* showed the largest RNA-seq response, with a log_2_ fold change of 5.68, followed by *SvPRX47* and *SvPRX61*, with log_2_ fold changes of 4.08 and 3.60, respectively. The remaining candidates—*SvPRX12*, *SvPRX41*, *SvPRX23*, and *SvPRX66*—showed log_2_ fold changes ranging from 1.25 to 3.11.

*SvPRX61* was expressed at a mean level of 33.55 TPM in CK roots and 406.48 TPM under Cd100 treatment. *SvPRX62* showed a lower basal abundance but a larger relative response, increasing from 4.05 TPM in CK to 207.72 TPM under Cd100 treatment (**Figure 4C**). Thus, both genes were strongly induced by Cd100, although they differed in basal expression and response magnitude.

### 3.5 Global DIA proteome quality and SvaPRX protein coverage

The DIA analysis identified 22,883 peptides and 6,068 proteins, with 5,511–5,741 proteins showing positive quantitative values in each sample (**Supplementary Figures S8A, B**). Abundance distributions were comparable across the six samples (**Supplementary Figure S8C**). Principal component analysis based on 4,849 proteins quantified in all samples separated CK and Cd100 primarily along PC1, which explained 52.6% of the variance, while PC2 explained 21.0% (**Supplementary Figure S8D**). A sensitivity PCA including 5,921 proteins quantified in at least three samples, with within-protein median imputation for missing log_2_ abundances, produced the same treatment separation along PC1; PC1 and PC2 explained 47.7% and 21.5% of the variance, respectively. Within-treatment Pearson correlations ranged from 0.949 to 0.960 for CK and from 0.949 to 0.967 for Cd100 (**Supplementary Figure S8E**). In addition, 75.3% and 74.4% of proteins had within-treatment coefficients of variation ≤30% in CK and Cd100, respectively (**Supplementary Figure S8F**). Together, these results supported the reproducibility of the global DIA dataset and indicated that treatment separation was not dependent on the complete-case protein subset.

Proteins corresponding to 40 of the 80 SvaPRX genes were quantified. Eleven SvaPRX proteins were significantly upregulated and six were significantly downregulated under Cd100 treatment. Quantification coverage and DEP proportions differed among phylogenetic groups (**Supplementary Figures S6A, B**). Gene-level protein measurements and their integration with RNA-seq results are provided in **Supplementary Table S10**.

### 3.6 Transcriptome-proteome integration and candidate prioritization

Integration of the RNA-seq and proteomic results separated the 80 genes into five observed evidence patterns. Seven genes showed concordant upregulation at the transcript and protein levels, 31 were identified as DEGs only, four were identified as differentially abundant proteins only, six displayed opposite response directions between the two molecular layers, and 32 lacked significant joint evidence. No gene showed concordant downregulation at both levels (**Figures 5A, B**; **Supplementary Figures S6C, D**).

**Figure 5:**
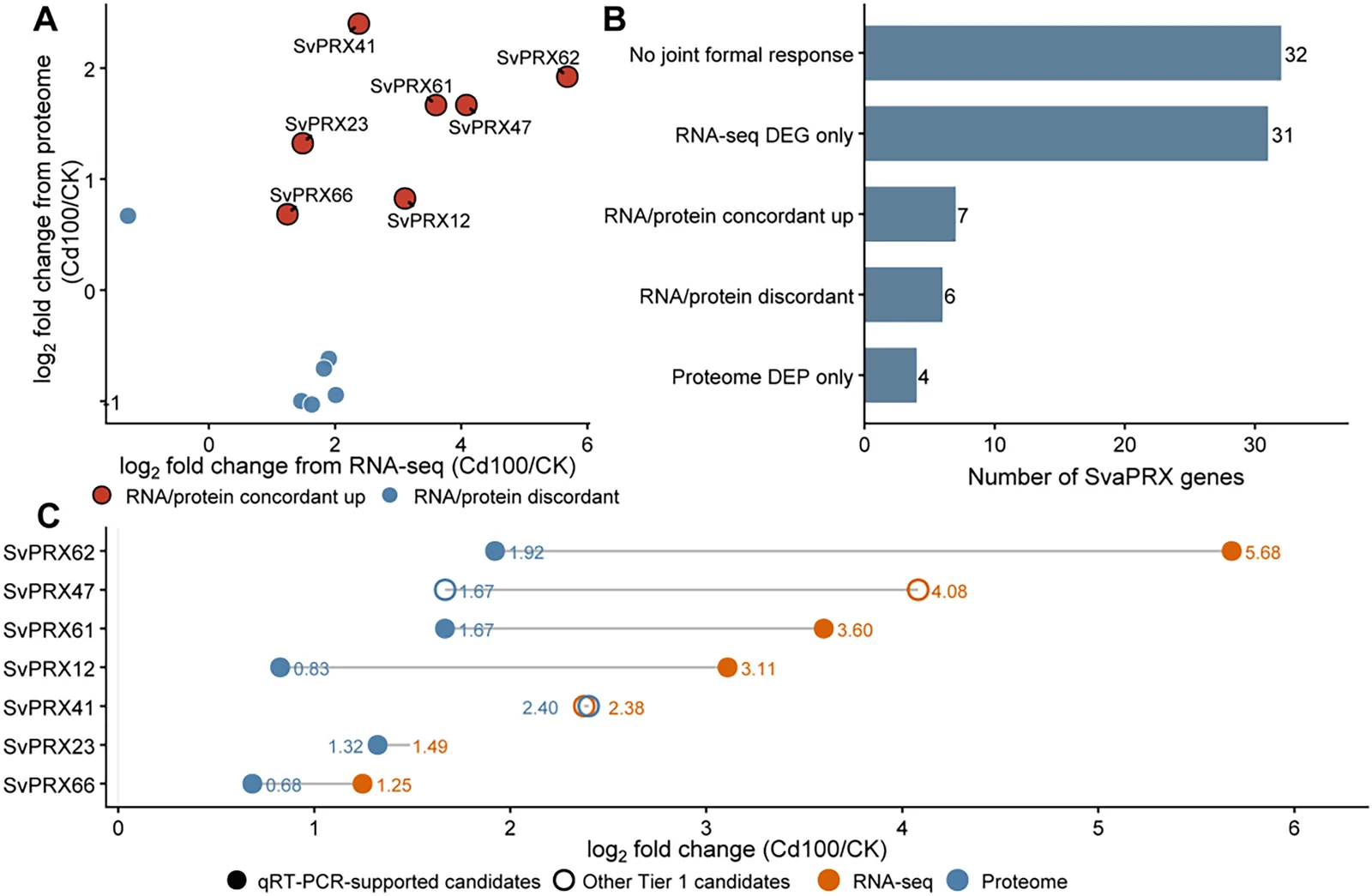
Cross-layer evidence integration and prioritization of SvaPRX genes under Cd100 treatment. **(A)** Comparison of RNA-seq and proteomic effect sizes for the 13 SvaPRX genes showing significant responses in both molecular layers. Filled points with black outlines indicate the qRT-PCR-supported candidates SvPRX61 and SvPRX62, whereas open points with black outlines indicate the other five Tier 1 candidates. **(B)** Numbers of SvaPRX genes assigned to each cross-layer evidence class. **(C)** RNA-seq and proteomic log_2_ fold changes of the seven Tier 1 candidates. Filled black symbols indicate the qRT-PCR-supported candidates, and open black symbols indicate the other Tier 1 candidates. The qRT-PCR results for SvPRX61 and SvPRX62 are presented separately in **Figure 6**.

Among the six genes with discordant responses, SvPRX08 and SvPRX15 (Other), SvPRX48 (C2), SvPRX67 (C3), and SvPRX78 (C8) were transcriptionally upregulated but showed reduced protein abundance. In contrast, SvPRX32 (C1) was transcriptionally downregulated but showed increased protein abundance. Thus, discordance occurred across four selected clades and the heterogeneous Other category rather than being concentrated in one phylogenetic group. These genes were retained in the full-family results but were not prioritized as directionally concordant candidates.

The seven genes showing concordant upregulation—*SvPRX12*, *SvPRX23*, *SvPRX41*, *SvPRX47*, *SvPRX61*, *SvPRX62*, and *SvPRX66*—were designated Tier 1 candidates. Their RNA-seq log_2_ fold changes ranged from 1.25 to 5.68, whereas the corresponding proteomic log_2_ fold changes ranged from 0.68 to 2.40 (**Figure 5C**). *SvPRX62* showed the largest transcript-level increase and a proteomic log_2_ fold change of 1.92. *SvPRX61* showed RNA-seq and proteomic log_2_ fold changes of 3.60 and 1.67, respectively.

*SvPRX41* had the lowest Cd100 transcript abundance among the Tier 1 candidates, with a mean of 1.74 TPM, but showed the largest proteomic effect size, with a log_2_ fold change of 2.40. Thus, absolute transcript abundance and relative protein response represented distinct dimensions of the integrated evidence. Because both transcript and protein abundance increased under Cd100 treatment, *SvPRX41* remained within the concordantly upregulated category rather than the discordant group.

The integrated candidate matrix further demonstrated variation in transcript abundance, RNA-seq and proteomic significance, promoter motif density, and motif diversity (**Supplementary Figure S7**). Cd100 transcript abundance ranged from 1.74 TPM for *SvPRX41* to 913.91 TPM for *SvPRX12*. Predicted promoter motif density ranged from 51.0 to 115.0 sites per kilobase, and the number of unique motifs ranged from 11 to 17. These features provided complementary descriptions of the candidates but did not identify a single gene that was consistently superior across every evidence type. The complete RNA-seq/proteome integration results, evidence classifications, and final candidate-priority categories for all 80 SvaPRX genes are provided in **Supplementary Table S10**.

### 3.7 Targeted qRT-PCR assessment of SvPRX61 and SvPRX62

To provide an independent expression-level assessment of the cross-omics prioritization, SvPRX61 and SvPRX62 were examined by qRT-PCR using three independent biological replicates. These genes were selected as representative high-priority candidates because SvPRX62 and SvPRX61 ranked first and third, respectively, in the reproducible Tier 1 display order, showed concordant transcript- and protein-level induction, represented different phylogenetic contexts, and yielded gene-specific, reproducible assays. Relative expression of SvPRX61 increased by approximately 4.83-fold under Cd100 treatment compared with CK (P = 0.00098). SvPRX62 showed a larger response, with an approximately 19.59-fold increase under Cd100 treatment (P = 0.00149; **Figure 6A**).

**Figure 6:**
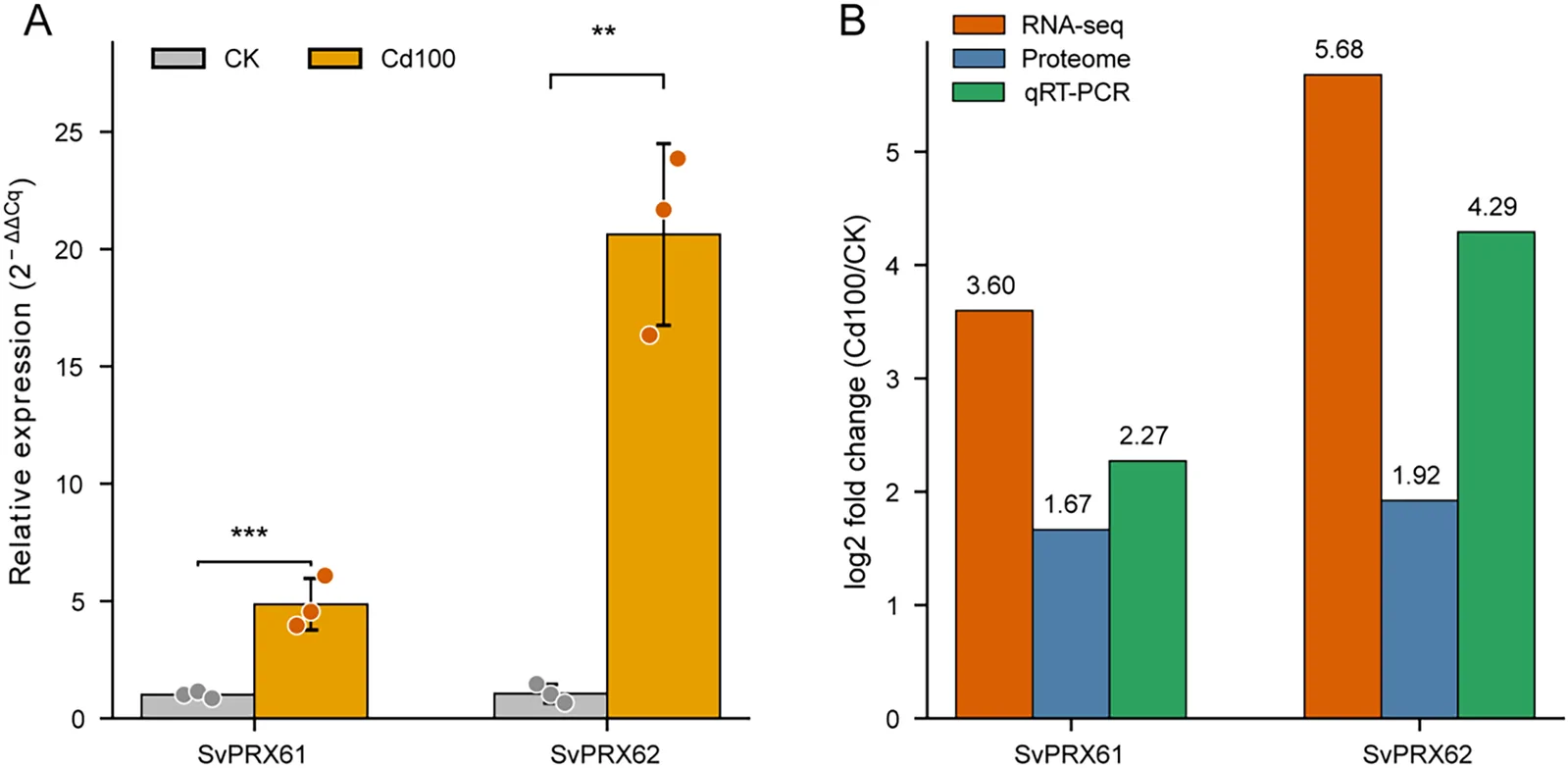
Targeted qRT-PCR assessment of SvPRX61 and SvPRX62 under cadmium treatment. **(A)** Relative expression levels of SvPRX61 and SvPRX62 in roots under CK and Cd100 conditions. Expression was normalized to the working reference gene TIP41 and calculated using the 2^-^ΔΔCq method. Bars show the mean ± SD of three biological replicates, and points represent individual biological replicates. Statistical comparisons were performed using biological-replicate ΔCq values; **P < 0.01 and ***P < 0.001. **(B)** Cross-layer comparison of log_2_ fold changes obtained from RNA-seq, proteomic, and qRT-PCR analyses for SvPRX61 and SvPRX62.

The qRT-PCR-derived log_2_ fold changes were 2.27 for *SvPRX61* and 4.29 for *SvPRX62*. For *SvPRX61*, the log_2_ fold changes obtained from RNA-seq, proteomic, and qRT-PCR analyses were 3.60, 1.67, and 2.27, respectively. The corresponding values for *SvPRX62* were 5.68, 1.92, and 4.29 (**Figure 6B**).

For both genes, qRT-PCR supported the direction of the RNA-seq response. Although effect-size estimates differed among platforms, RNA-seq, proteomic, and qRT-PCR measurements all indicated induction under Cd100 treatment. Together, these results provide additional directional expression support for SvPRX61 and SvPRX62. They do not constitute validation of the remaining five Tier 1 candidates or demonstrate physiological functions in Cd tolerance.

## 4 Discussion

### 4.1 Expansion and structural diversification of the SvaPRX family

Class III peroxidases constitute large and evolutionarily dynamic gene families in land plants, and their family sizes vary among species as a result of lineage-specific duplication, retention, and gene loss (Tognolli et al., 2002; Welinder et al., 2002; Duroux and Welinder, 2003; Passardi et al., 2004). In the present study, 80 high-confidence class III peroxidase genes were identified in *Salix variegata*, slightly more than the 73 members reported in *Arabidopsis thaliana*. This family size was also comparable to the 93 class III peroxidase genes identified in *Populus trichocarpa*, a closely related member of the Salicaceae, providing a more direct evolutionary reference for the number of SvaPRX members (Ren et al., 2014). Class III peroxidase families of similar or larger sizes have also been reported in rice and Chinese pear (Passardi et al., 2004; Cao et al., 2016).

All 80 SvPRX genes were assigned to segmental, dispersed, tandem, or proximal duplication categories, with segmental and dispersed duplicates accounting for most family members. Direct interchromosomal collinearity was also detected among several SvaPRX loci. These findings indicate that both large-scale and local duplication contributed to the expansion of the family. A complex combination of whole-genome duplication, tandem duplication, gene loss, and chromosomal rearrangement has similarly shaped the class III peroxidase family in Populus (Ren et al., 2014). We did not calculate Ka/Ks ratios because MCScanX duplication-origin categories do not by themselves establish unambiguous pairwise evolutionary histories. Robust selection inference would require curated codon alignments, evaluation of synonymous-site saturation, and preferably cross-species syntenic anchors. These analyses were outside the family-identification and cross-omics-prioritization scope of the present study; consequently, no claims are made here about duplication age or selective pressure on individual pairs.

Despite this expansion, the family retained substantial structural conservation. Most SvPRX genes contained three or four exons, and several protein motifs were detected in nearly all members. The almost universal occurrence of Motifs 2, 4, 6, and 7 suggests that these motifs form part of the conserved structural core required for class III peroxidase folding and catalytic activity. Nevertheless, the biochemical roles of the individual MEME motifs were not directly assigned in this study. Variation in the remaining motifs, exon–intron organization, and protein length may reflect sequence diversification after gene duplication and could contribute to functional specialization among family members (Welinder et al., 2002; Passardi et al., 2004; Ren et al., 2014; Cao et al., 2016).

The C1–C8 groups were defined from the topology of the combined *S. variegata*–*A. thaliana* phylogenetic tree and should therefore be regarded as study-specific phylogenetic units rather than established functional subclasses. The 34 proteins placed in the Other category did not constitute a low-confidence group; instead, they represented a heterogeneous set that did not fall within the eight selected clades. All of these proteins were retained in the subsequent structural and expression analyses. Importantly, this category included *SvPRX62*, one of the most strongly Cd-responsive genes identified in the present study. Thus, phylogenetic position provided a useful evolutionary framework but was insufficient on its own to predict Cd responsiveness or biological function.

### 4.2 Potential links with apoplastic redox regulation and cell-wall processes

Most SvaPRX proteins were predicted to be extracellular, and a large proportion showed combined sequence support for secretion and extracellular localization. This pattern agrees with the general properties of class III peroxidases, which are commonly transported to the apoplast, cell wall, or vacuolar system (Shigeto and Tsutsumi, 2016; Cosio and Dunand, 2009; Francoz et al., 2015). In the apoplast, these enzymes can consume hydrogen peroxide while oxidizing phenolic substrates involved in lignin polymerization and cell-wall cross-linking. Depending on the catalytic cycle, substrate availability, and cellular context, individual class III peroxidases may also contribute to ROS production and processes associated with cell-wall loosening (Shigeto and Tsutsumi, 2016; Cosio and Dunand, 2009; Francoz et al., 2015). Functional studies in Arabidopsis further demonstrate that different PRX members can exert distinct effects on ROS distribution, cell expansion, and root development (Raggi et al., 2015; Marzol et al., 2022; Pacheco et al., 2022).

Cd100 treatment induced a broad transcriptional response across the SvaPRX family. Forty-two genes were upregulated, whereas only two genes were downregulated under the applied differential-expression criteria. All members of C7 and C8 were transcriptionally induced, although the small sizes of these groups require cautious interpretation of their response proportions. Overall, the predominance of upregulated genes indicates that transcriptional activation was the major family-wide response to Cd100 treatment.

Previous investigations in willow have linked Cd responses to metal transport, antioxidant regulation, phenylpropanoid metabolism, cell-wall modification, and lignin biosynthesis (Shi et al., 2016; Yang et al., 2015; Yu et al., 2023). In *Salix matsudana*, combined morphological, transcriptomic, and proteomic evidence showed that Cd accumulated extensively in root cell walls and that Cd exposure was accompanied by increased lignin accumulation and activation of lignin-biosynthetic genes and proteins (Yu et al., 2023). These observations provide a biologically plausible context for the broad induction of SvaPRX genes. Nevertheless, the present expression data only support the potential participation of SvaPRX members in Cd-responsive apoplastic and cell-wall processes. They do not establish whether individual enzymes promote wall stiffening, wall loosening, ROS consumption, or other specific biochemical reactions.

Considerable differences in predicted promoter-motif composition were observed among the SvPRX genes, which may provide a sequence basis for differential transcriptional responses among duplicated family members. DOF3.6 and AHL13 were the most frequently detected predicted motifs and occurred broadly across the family. Their widespread distribution, however, does not demonstrate Cd-specific regulation. In the absence of background enrichment analysis and experimental binding evidence, the promoter results are best interpreted as descriptive regulatory context rather than direct evidence that particular transcription factors control the observed Cd responses.

### 4.3 Proteome quality and cross-layer prioritization

The global DIA quality assessment showed comparable abundance distributions, high within-treatment correlations, and treatment separation in principal component space. Importantly, the same separation was recovered in a sensitivity analysis that retained 5,921 proteins quantified in at least three samples, indicating that the primary PCA result was not dependent on restricting the analysis to the 4,849 complete-case proteins. These observations strengthen confidence that the SvaPRX-level protein changes were evaluated within a reproducible proteomic dataset rather than inferred from isolated protein records (**Supplementary Figure S8**).

Transcriptomic analysis identified numerous Cd-responsive SvPRX genes, whereas the proteomic data showed that changes in transcript and protein abundance were not uniformly coupled. Seven genes showed significant and directionally concordant upregulation at both molecular levels. In contrast, SvPRX08, SvPRX15, SvPRX48, SvPRX67, and SvPRX78 were transcriptionally upregulated but showed reduced protein abundance, whereas SvPRX32 showed the opposite pattern. These six members were distributed across C1, C2, C3, C8, and Other, providing no evidence that discordance was restricted to a single phylogenetic lineage.

Differences between transcriptomic and proteomic responses can arise from delayed translation, transcript-specific translational control, altered mRNA stability, miRNA-mediated regulation, selective protein degradation, secretion or trafficking, and technical differences in molecular detection (Kosová et al., 2011). Protein-record multiplicity may also contribute to uncertainty for SvPRX48, for which three significant protein records mapped to the same gene; the remaining discordant genes each had one mapped significant protein record. Because both omics layers were measured at one 10-d endpoint and the study did not include small-RNA sequencing, mRNA-decay measurements, ribosome profiling, or protein-turnover assays, the present data cannot distinguish temporal offsets from these post-transcriptional and protein-stability mechanisms. Integrated transcriptomic and proteomic analysis has similarly refined Cd-responsive pathways and candidates in Sedum plumbizincicola, where only a subset of transcript changes was accompanied by corresponding protein-level responses (Zhu et al., 2022). The discordant members are therefore retained as biologically informative follow-up targets rather than treated as errors, but they were not ranked as concordant candidates.

The 31 genes supported only by RNA-seq should not be considered biologically irrelevant. Protein-level changes may have remained below the detection limit, or transcript induction may not have resulted in detectable protein accumulation because of post-transcriptional regulation. However, because these genes lacked formal proteomic support, they were assigned a lower priority in the present cross-layer screening framework. Thus, the integrated analysis was used to prioritize candidates rather than to exclude all other Cd-responsive genes from potential biological importance.

The candidate profiles also demonstrate why no single measurement was sufficient for prioritization. *SvPRX41*, for example, had the lowest Cd100 transcript abundance among the seven Tier 1 candidates but showed the largest protein-level fold change. These findings are not contradictory because transcript abundance under one condition and the relative protein difference between treatments represent distinct dimensions of the response. The pattern may indicate regulation beyond absolute transcript abundance, although the underlying process cannot be distinguished from the present data.

Similarly, the seven Tier 1 genes differed in basal transcript abundance, RNA and protein effect sizes, statistical significance, promoter motif density, and motif diversity. Formal candidate designation was therefore based on concordant transcript and protein responses, whereas promoter characteristics were retained as supporting contextual information. Avoiding a single weighted score prevented predicted promoter features from being assigned the same evidential value as experimentally measured expression changes.

### 4.4 SvPRX61 and SvPRX62 as qRT-PCR-supported Cd-responsive candidates

Targeted qRT-PCR analysis provided additional support for Cd-induced expression of SvPRX61 and SvPRX62. Although the qRT-PCR-derived log_2_ fold changes were lower than the corresponding RNA-seq estimates, both approaches showed the same response direction, and protein abundance also increased for both genes. The agreement in direction across the three datasets supports their classification as Cd-responsive candidates, while the single working reference gene and two-gene scope require cautious interpretation and do not demonstrate physiological functions in Cd tolerance.

The two genes exhibited different response characteristics. *SvPRX61* had a higher transcript abundance under both CK and Cd100 conditions, whereas *SvPRX62* showed a lower basal expression level but a larger relative induction. They were also located in different phylogenetic contexts: *SvPRX61* was assigned to C7, whereas *SvPRX62* belonged to the heterogeneous Other category. These findings indicate that strongly Cd-responsive members were not restricted to a single phylogenetic group.

Previous studies have demonstrated that individual class III peroxidases can regulate ROS distribution, root development, cell-wall cross-linking, and lignification (Raggi et al., 2015; Marzol et al., 2022; Pacheco et al., 2022; Kidwai et al., 2019). For example, Arabidopsis PRX62 and PRX69 participate in root-hair responses to low temperature, whereas rice *OsPRX38* has been associated with enhanced apoplastic lignification and reduced arsenic accumulation (Pacheco et al., 2022; Kidwai et al., 2019). These studies illustrate the functional diversity of stress-responsive class III peroxidases but do not establish equivalent functions for the willow candidates. In particular, the shared numerical designation of *SvPRX62* and Arabidopsis PRX62 does not indicate orthology; no one-to-one orthologous relationship between these proteins was established in the present study.

Accordingly, the present evidence supports describing SvPRX61 and SvPRX62 as qRT-PCR-supported Cd-responsive candidates rather than genes with demonstrated functions in Cd resistance. The targeted assay did not test the other five Tier 1 candidates, and the broader experimental limitations and priorities for future validation are summarized below.

### 4.5 Conclusions and study limitations

This study identified 80 high-confidence class III peroxidase genes in S. variegata and combined genomic characterization with RNA-seq and DIA proteomic evidence. Cd100 induced broad transcriptional activation across the family, whereas formal transcript–protein concordance was restricted to seven candidates from different phylogenetic contexts. SvPRX61 and SvPRX62 received additional directional support from targeted qRT-PCR analysis and therefore represent focused candidates for subsequent investigation. Overall, this study establishes a family-specific cross-omics framework for prioritizing Cd-responsive PRXs but does not assign physiological functions to individual genes.

Several limitations define the scope of these conclusions. The study was conducted under a controlled artificial-light regime lower than full natural sunlight and used a single, relatively high Cd concentration (100 μM) and one treatment duration (10 d). These conditions were intended to provide a uniform hydroponic environment and a clearly detectable root molecular response rather than to simulate field irradiance or typical dissolved Cd concentrations in natural environments. Consequently, the present design cannot resolve potential interactions between light intensity and Cd stress, response thresholds, dose dependence, temporal dynamics, or candidate behavior under lower and more environmentally representative Cd exposure. In addition, promoter and subcellular-localization results were based on computational predictions, qRT-PCR analysis used a single working reference gene and was restricted to SvPRX61 and SvPRX62, and no candidate-specific enzyme-activity, protein-localization, cell-wall-composition, or genetic-manipulation experiments were performed. Future studies combining multiple light regimes, Cd concentrations, and sampling times with validated multiple-reference-gene qPCR, biochemical, cellular, and genetic analyses will be required to determine the physiological functions of the prioritized candidates.

## Data Availability

The raw RNA-seq data generated in this study are publicly available in the Genome Sequence Archive (GSA) at the National Genomics Data Center under accession CRA040508 (https://ngdc.cncb.ac.cn/gsa/browse/CRA040508), associated with BioProject PRJCA060820 (https://ngdc.cncb.ac.cn/bioproject/browse/PRJCA060820). The mass spectrometry proteomics data have been deposited with the ProteomeXchange Consortium via the iProX partner repository (Ma et al., 2019; Chen et al., 2022) under dataset identifier PXD082203 (https://www.iprox.cn/page/project.html?id=IPX0018836000). The processed data supporting the conclusions of this article are included in the article and its **Supplementary Material**.
