# Supplementary material for: Integrated genomic, transcriptomic, and proteomic analyses prioritize cadmium-responsive class III peroxidases in Salix variegata
Source: Front Plant Sci. 2026 Sep 28;17:1956650. doi: 10.3389/fpls.2026.1956650 (PMC13655147; doi:10.3389/fpls.2026.1956650)
Supplement: Supplementary file 1 [file Supplementaryfile1.docx]

**Figure S1.** Workflow for identification and curation of the SvaPRX family and downstream analyses. Arabidopsis class III PRX proteins were used as queries against the S. variegata proteome, followed by domain-based screening and integrated manual curation. The final set of 80 high-confidence SvaPRX proteins was used for phylogenetic, chromosomal-distribution, gene-structure, promoter-motif, transcriptomic, and proteomic analyses.

**Figure S2.** Protein properties and predicted localization evidence for the SvaPRX family. (A) Distribution of protein lengths; the dashed line indicates the median of 328 aa. (B) Relationship between molecular weight and theoretical pI; selected proteins with atypical properties are labeled, and point colors indicate DeepTMHMM classes: signal peptide (SP), globular (GLOB), or transmembrane (TM). (C) DeepLoc-predicted subcellular localization categories. (D) Integrated extracellular/secretory evidence classes based on SignalP, DeepLoc, DeepTMHMM, and manual review.

**Figure S3.** Complete gene structure and conserved motif organization of all 80 SvaPRX members. Exon–intron structures are shown on the left, and the positions of conserved MEME motifs are shown on the right, using the same gene order.

**Figure S4.** Promoter sequence quality and JASPAR/FIMO motif characteristics of the SvaPRX family. (A) Lengths of the analyzed promoter sequences ordered by phylogenetic group; SvPRX80 is highlighted because its retrieved promoter was shorter than 2 kb. (B) Distribution of retained FIMO hits per kilobase among phylogenetic groups. (C) Distribution of the number of unique JASPAR motifs per promoter among phylogenetic groups. (D) The ten motifs with the highest numbers of retained FIMO hits (q ≤ 0.05); labels indicate the number of promoters containing each motif.

**Figure S5.** RNA-seq quality control and full-family expression patterns of SvaPRX genes. (A) Pearson correlation matrix among the six root RNA-seq libraries. (B) Row-wise Z-score heatmap of log₂(TPM + 1) values for all 80 SvaPRX genes. Side annotations indicate phylogenetic group, RNA-seq response class, and final candidate category: qRT-PCR-supported candidates, other Tier 1 candidates, or other genes. (C) Numbers of genes classified as Cd-upregulated DEGs, Cd-downregulated DEGs, or not identified as DEGs in the CK-versus-Cd100 comparison.

**Figure S6.** Proteomic support and RNA/protein response patterns across the SvaPRX family. (A) Proportions of quantified and non-quantified SvaPRX proteins within each phylogenetic group. (B) Proportions of upregulated DEPs, downregulated DEPs, and proteins not identified as significant DEPs within each phylogenetic group. (C) Relationship between proteomic log₂ abundance change and log₁₀(Cd100 mean TPM + 0.1) for SvaPRX proteins with proteomic measurements. Point colors indicate phylogenetic groups. Filled points with black outlines indicate SvPRX61 and SvPRX62, whereas open points with black outlines indicate the other five Tier 1 candidates. (D) Distribution of RNA/protein evidence classes among phylogenetic groups.

**Figure S7.** Integrated evidence for the seven Tier 1 SvaPRX candidates. (A) Ranked candidate list. Point fill distinguishes the qRT-PCR-supported candidates SvPRX61 and SvPRX62 from the other five Tier 1 candidates, and labels show phylogenetic group and duplication type. (B) Scaled evidence matrix integrating RNA-seq effect size, proteomic effect size, Cd100 transcript abundance, RNA-seq and proteomic significance, FIMO hit density, and the number of unique motifs. Numerical labels in the cells show the corresponding unscaled source values. (C) Relationship between promoter FIMO hit density and Cd100 transcript abundance for the seven candidates; marker categories are the same as in panel A.

**Figure S8.** Quality assessment of the DIA proteomic dataset. (A) Total numbers of identified peptides and proteins. (B) Numbers of proteins quantified with positive abundance values in each biological replicate. (C) Distributions of log₂-transformed positive protein abundances across the six samples. Boxes indicate the median and interquartile range, and whiskers extend to 1.5 times the interquartile range. (D) Principal component analysis based on 4,849 proteins quantified in all six samples. The percentages of variance explained by PC1 and PC2 are shown on the axes. (E) Pairwise Pearson correlation matrix of log₂-transformed protein abundances; each sample pair was analyzed using proteins quantified in both samples. (F) Empirical cumulative distributions of within-group coefficients of variation (CVs), calculated using proteins quantified in all three biological replicates of CK or Cd100. The dashed and dotted vertical lines indicate CV thresholds of 20% and 30%, respectively. Gray and orange indicate CK and Cd100, respectively.
